# An Artificial Intelligence based model for predicting long-term all-cause mortality after acute Myocardial Infarction (the AIMI model)

**DOI:** 10.1093/ehjdh/ztag078

**Published:** 2026-05-20

**Authors:** Linghan Xue, Wenmiao Wang, Qianli Zhao, Wentao Li, Wenhao Dong, Shaodi Yan, Xiaoxiao Zhao, Jiannan Li, Runzhen Chen, Nan Li, Shuai He, Chen Liu, Peng Zhou, Yi Chen, Li Song, Hongbing Yan, Zhi Liu, Hanjun Zhao

**Affiliations:** Department of Cardiology, Fuwai Hospital, National Center for Cardiovascular Diseases, Peking Union Medical College and Chinese Academy of Medical Sciences, No. 167 North Lishi Road, Xicheng District, Beijing 100037, China; Department of Thoracic Surgery, The Second Qilu Hospital of Shandong University, Shandong University, Jinan, Shandong, China; MS Information Systems and Artificial Intelligence for Business Program, Johns Hopkins Carey Business School, Washington D.C., USA; School of Software, Shandong University, Jinan, Shandong, China; Department of Cardiology, Fuwai Hospital, National Center for Cardiovascular Diseases, Peking Union Medical College and Chinese Academy of Medical Sciences, No. 167 North Lishi Road, Xicheng District, Beijing 100037, China; Fuwai Hospital, Chinese Academy of Medical Sciences, Shenzhen, China; Department of Cardiology, Fuwai Hospital, National Center for Cardiovascular Diseases, Peking Union Medical College and Chinese Academy of Medical Sciences, No. 167 North Lishi Road, Xicheng District, Beijing 100037, China; Department of Cardiology, Fuwai Hospital, National Center for Cardiovascular Diseases, Peking Union Medical College and Chinese Academy of Medical Sciences, No. 167 North Lishi Road, Xicheng District, Beijing 100037, China; Department of Cardiology, Fuwai Hospital, National Center for Cardiovascular Diseases, Peking Union Medical College and Chinese Academy of Medical Sciences, No. 167 North Lishi Road, Xicheng District, Beijing 100037, China; Department of Cardiology, Fuwai Hospital, National Center for Cardiovascular Diseases, Peking Union Medical College and Chinese Academy of Medical Sciences, No. 167 North Lishi Road, Xicheng District, Beijing 100037, China; Department of Cardiothoracic Surgery, Affiliated Hospital of Nantong University, Nantong, China; Department of Cardiology, Fuwai Hospital, National Center for Cardiovascular Diseases, Peking Union Medical College and Chinese Academy of Medical Sciences, No. 167 North Lishi Road, Xicheng District, Beijing 100037, China; Department of Cardiology, Fuwai Hospital, National Center for Cardiovascular Diseases, Peking Union Medical College and Chinese Academy of Medical Sciences, No. 167 North Lishi Road, Xicheng District, Beijing 100037, China; Department of Cardiology, Fuwai Hospital, National Center for Cardiovascular Diseases, Peking Union Medical College and Chinese Academy of Medical Sciences, No. 167 North Lishi Road, Xicheng District, Beijing 100037, China; Department of Cardiology, Fuwai Hospital, National Center for Cardiovascular Diseases, Peking Union Medical College and Chinese Academy of Medical Sciences, No. 167 North Lishi Road, Xicheng District, Beijing 100037, China; Department of Cardiology, Fuwai Hospital, National Center for Cardiovascular Diseases, Peking Union Medical College and Chinese Academy of Medical Sciences, No. 167 North Lishi Road, Xicheng District, Beijing 100037, China; Department of Cardiology, Beijing Amcare Hospital, No. 111 Jingshun Road, Chaoyang District, Beijing 100028, China; School of Information Science and Engineering, Shandong University, Qingdao, 72 Binhai Road, Jimo District, Qingdao, Shandong 266237, China; Department of Cardiology, Fuwai Hospital, National Center for Cardiovascular Diseases, Peking Union Medical College and Chinese Academy of Medical Sciences, No. 167 North Lishi Road, Xicheng District, Beijing 100037, China; Coronary Heart Disease Center, Fuwai Hospital, Chinese Academy of Medical Sciences, No. 167 North Lishi Road, Xicheng District, Beijing 100037, China

**Keywords:** Acute myocardial infarction, Mortality, Prediction model, Risk assessment, Machine learning

## Abstract

**Aims:**

Predicting long-term mortality after acute myocardial infarction (AMI) remains challenging. We aimed to establish an Artificial Intelligence—based model for predicting long-term all-cause mortality after AMI (the AIMI model).

**Methods and results:**

AIMI model was employed by RF (Random forest). Individual predictions were visualized by SHAP plots. AIMI model was compared against existing clinical risk scores using time-dependent ROC (receiver operating characteristic) curves, and Kaplan-Meier (K-M) analyses. External validation was also performed at the same way. Brier scores were calculated in validation cohorts. We consecutively enrolled 4825 AMI patients underwent emergent coronary angiography or PCI procedures within 24 h of symptom onset to train and test the AIMI model and 723 AMI patients for external validation. Model incorporated 15 variables achieved robust performance (C-index = 0.81). As indicated by AUCs in the test set, AIMI model outperformed GRACE and TIMI risk scores across short-, mid- and long-term periods, especially for long-term prediction (1, 3 and 5 years). K-M curves confirmed precise discrimination between low-, median-, and high-risk groups (all *P* < 0.05). External validation confirmed good generalization and robustness for AIMI model (AUCs: 0.88, 0.91, 0.83, 0.75, 0.78 and 0.77 during hospitalization, at 30 days, half-year, 1-year, 2-years and 3-years follow-up; comparisons of K-M curves across three risk groups, all *P* < 0.05). Brier scores demonstrated good individual level performance (internal validation cohort: 0.022; external validation: 0.021).

**Conclusion:**

The AIMI model surpassed traditional methods for long-term all-cause death prediction after AMI. AI-based model demonstrated potential to enhance risk stratification and guide post-discharge management.

## Graphical Abstract

**Graphical Abstract:**
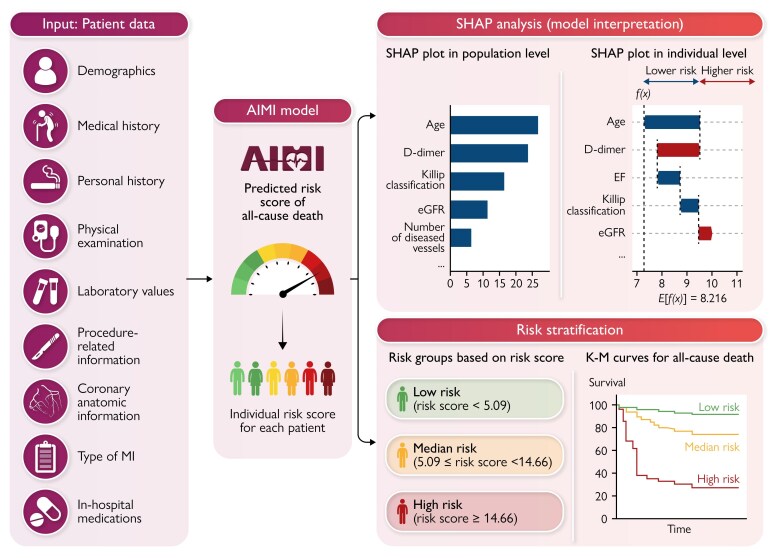

## Introduction

Acute myocardial infarction (AMI) remains a major global public health challenge, contributes significantly to morbidity and mortality.^1^ Despite significant progress has been made in the management of AMI over the past few decades, patients are still faced with higher in-hospital and post-hospital mortality.^2^ The Global Registry of Acute Coronary Events (GRACE) and the Thrombolysis in Myocardial Infarction (TIMI) scores are widely used models to estimate short-term mortality for AMI.^3,4^ However, such conventional risk models established by classical statistic methods, without considering interactions between variables, non-linear and time-dependent influences on outcomes, and thus offer limited power to predict long-term mortality.^5–8^ Machine learning (ML) overcomes those limitations and provides more efficient, faster, and personalized clinical practice as it explores large amounts of information automatically and systematically,^8,9^ thus may bring opportunities to improve long-term risk stratification for AMI.

This study aims to give personalized weights of risk factors for individual patients and develop an individualized risk model for all-cause death prediction across short-, mid- and long-term period by ML-based algorithm, thus achieves more precise risk stratification.

## Patients and methods

### Study population

The study is multi-centre observational study. The locations of two centres are marked on Chinese map in Supplementary material online, *Figure S1*. AMI patients who underwent emergent coronary angiography or percutaneous coronary intervention (PCI) procedures within 24 h of symptom onset between January 2010 and January 2020 from *Fuwai Hospital, National Centre for Cardiovascular Diseases, Chinese Academy of Medical Sciences and Peking Union Medical College, Beijing, China* were consecutively enrolled for training and testing. Exclusion criteria included incomplete baseline data and missing follow-up data. The samples in the external cohort were collected in another hospital from a more recent period. Patients with AMI were consecutively recruited between May 2019 and April 2021 from *Fuwai Hospital, Chinese Academy of Medical Sciences, Shenzhen, Guangdong province, China,* where was 1940 km from Beijing. The cohorts from two centres are entirely distinct with no overlap. In the external validation cohort, no patients were excluded, all patients had complete follow-up data, and missing baseline data were filled by RF algorithm.

The study protocol adhered to the tenets of the Declaration of Helsinki. It was approved by the Ethics Review Board of Fuwai Hospital & National Centre for Cardiovascular Diseases (approval number 2016-I2M-1-009, 2017-866 and 2025-2663) and the Ethics Review Board of Shenzhen Clinical Research Centre for Cardiovascular Diseases, *Fuwai Hospital, Chinese Academy of Medical Sciences, Shenzhen* [approval number SP2021085 (01)]. The Written informed consent was obtained from all patients.

### Follow-up and outcome

Follow-up was conducted regularly for all enrolled patients, with the study endpoint defined as all-cause mortality. The first follow-up at one month was performed during outpatient clinic visits, where patients underwent comprehensive clinical examinations. For patients unable to return to the clinic, follow-up was conducted via telephone interviews by staff from the information centre using a standardized questionnaire. Subsequent follow-ups were carried out at six months and twelve months via telephone. For patients who survived beyond one year, annual follow-up was scheduled in subsequent years. All-cause death was confirmed by contacting family members and was verified using death certificates.

### Data collection

In total, 42 features at 9 aspects were collected, including demographics, medical history, personal history, physical examination, laboratory values, procedure-related information, coronary anatomic information, in-hospital medications and the type of MI. Demographics included age, sex, and body mass index (BMI). Medical history included atrial fibrillation (AF), hypertension, hyperglycemia, diabetes, chronic kidney disease (CKD), previous percutaneous coronary intervention (PCI) and previous coronary artery bypass grafting (CABG). Personal history included smoking. Physical examination included heart rate, systolic blood pressure (SBP), diastolic blood pressure (DBP), ejection fraction (EF) and Killip classification. Laboratory values included triglyceride (TG), high-density lipoprotein cholesterol (HDL-C), low-density lipoprotein cholesterol (LDL-C), lipoprotein (a) [Lp(a)], creatinine, estimated glomerular filtration rate (eGFR), high-sensitivity C-reactive protein (hs-CRP) and D-dimer. Procedure-related information included pre-procedure TIMI flow, post-procedure TIMI flow, stent implantation, thrombus aspiration and intra-aortic balloon pumping (IABP). Coronary anatomic information included number of diseased vessels, culprit vessel [left main coronary artery (LM), left anterior descending artery (LAD), left circumflex artery (LCX), right coronary artery (RCA) or bypass graft], reference diameter, lesion length, diameter stenosis and American Heart Association (AHA) classification of culprit lesion. In-hospital medications included aspirin, clopidogrel, ticagrelor, ACEI/ARB/ARNI, beta-blockers and statin. The type of myocardial infarction indicates a diagnosis of ST-segment elevation myocardial infarction (STEMI) or non-ST-segment elevation myocardial infarction (NSTEMI). Values of laboratory and physical examination are all based on the first measurement taken after patients’ arrival or admission (*Table 1*).

**Table 1:** Forty-two predictive variables

**Patient demographics**	22. Estimated Glomerular Filtration Rate (eGFR) (mL/min/1.73 m^2^)
1. Age	23. High-Sensitivity C-Reactive Protein (hs-CRP) (mg/L)
2. Sex	24. D-dimer (μg/mL)
3. BMI	**Procedure-related Information**
**Medical history**	25. Pre-procedure TIMI flow
4. Atrial Fibrillation (AF)	26. Post-procedure TIMI flow
5. Hypertension	27. Stent implantation
6. Hyperglycemia	28. Thrombus Aspiration
7. Diabetes	29. IABP
8. Chronic kidney disease (CKD)	**Coronary anatomic information**
9. Previous PCI	30. Number of diseased vessels
10. Previous CABG	31. Culprit vessel
**Personal history**	32. Reference diameter
11. Smoking	33. Lesion length
**Physical examination**	34. Diameter stenosis
12. Heart rate	35. AHA Classification
13. Systolic blood pressure (SBP)	**In-hospital medications**
14. Diastolic blood pressure (DBP)	36. Aspirin
15. Ejection Fraction (EF)	37. Clopidogrel
16. Killip classification	38. Ticagrelor
**Laboratory values**	39. ACEI/ARB/ARNI
17. Triglyceride (TG) (mmol/L)	40. beta-blockers
18. High-Density Lipoprotein Cholesterol (HDL) (mmol/L)	41. Statin
19. Low-Density Lipoprotein Cholesterol (LDL) (mmol/L)	**Type of MI**
20. Lipoprotein (a) [Lp(a)](mg/L)	42. STEMI/NSTEMI
21. Creatinine (μmol/L)	

Abbreviations: BMI, body mass index; CABG, coronary artery bypass grafting; IABP, intra-aortic balloon pump; NSTEMI, non-ST-segment elevation myocardial infarction; PCI, percutaneous coronary intervention; STEMI, ST-segment elevation myocardial infarction.

### Machine learning models

We used 5 survival ML algorithms: Random Forest (RF), Survival Support Vector Machine (SSVM), Cox Proportional Hazard (CoxPH), Gradient Boosting with Stochastic Averaging (GBSA), and extreme gradient boosting (XGB). These algorithms were implemented in Python using the following packages: scikit-survival package for the construction of RF, SSVM and GBSA models, lifelines package for CoxPH model and xgboost package for XGB model. All of these methods have been modified previously into survival regression models capable of handling right-censored survival data.^10^ The labels included both the all-cause death status (whether or not all-cause death is observed during the follow-up period) and the survival time information (time at which all-cause death occurs). Risk scores of patients were calculated by five machine learning algorithms and then they were segmented into three risk groups using K-means clustering algorithm with elbow method.^11^ All ML models were systematically configured to employ the selected features, aiming to stratify AMI patients into high-, median- and low-risk groups.

### Model construction and evaluation

#### Data set division

The whole cohort from Beijing was divided in a 7:3 ratio. This division allocated seven parts for training, utilizing the remaining three parts for internal testing. To ensure that the training and test sets had similar baseline distribution, we performed multiple random partitions of the data, and verified that there were no statistically significant differences in baseline characteristics between the groups. Comparisons were performed among the AIMI model, TIMI and GRACE (version 2.0) risk scores in the test cohort. TIMI (0-2 low risk; 3–4 intermediate risk; 5–7 high risk) and GRACE (version 2.0, <109 low risk; 109–140 intermediate risk; >140 high risk) risk scores were calculated for all patients (https://www.mdcalc.com/). Patients from Shenzhen, China, were enrolled in external validation cohort.

#### Feature selection

For feature selection, we employed recursive feature elimination (RFE) approach based on the RF algorithm, which was effective for classification tasks.^12^ RFE enhances predictive model performance by preventing overfitting while simultaneously improving generalization capability.^13^ We sequentially eliminated features with minimal impact on the outcome, iteratively refitting the models with progressively smaller feature sets.^14^ RFE was continued until a significant drop in model performance was observed. The selected features were then incorporated into five ML models for training and evaluation.

#### SHapley additive exPlanation analysis

To enhance the interpretability of the black-box model, we introduced SHapley Additive exPlanation (SHAP) analysis.^15^ SHAP values represent the contribution of each variable to the outcome. Colour gradient represents SHAP value magnitude and horizontal position indicates the directional effect on outcome.^13^ The SHAP value of a variable can be positive or negative, which indicates an increased or decreased likelihood of all-cause death. In our study, SHAP analysis plays a key role in 3 aspects: (ⅰ) precisely identifies the relevant features, aiding to feature selection and model optimization; (ⅱ) facilitates the determination of optimal clinical thresholds for continuous variables, thereby mitigating the impact of outliers, and simplifying the model while maintaining predictive performance; (ⅲ) reveals the overall importance of variables at the population level and directly indicates a variable's effect on all-cause death at the individual level.

#### Internal and external model evaluation

The performance of the model was evaluated by Kaplan-Meier (K-M) curves with multivariate log-rank test and also evaluated by time-dependent receiver operating characteristic (ROC) curves and calculated the areas under the ROC curves (AUC). The accuracy of prediction at a patient level was calculated by Brier score. Equally, external validation cohort was also evaluated with time-dependent ROC curves, Brier score calculations and K-M curves.

### Statistical analyses

Statistical analyses were performed using R (version 4.4.2) and Python (version 3.10.10). Categorical variables are presented as numbers (%) and were analyzed using Pearson’s chi-square test or Fisher’s exact test. Continuous variables were presented as mean ± standard deviation ($\bar{x}$±SD) for normally distributed data or median (interquartile range) [M(Q1-Q3)] for non-normally distributed data. Group comparisons were performed using Student’s *t*-test (normal distribution) or the Mann–Whitney U-test (non-normal distribution). Predictive performance was evaluated using C-index and time-dependent ROC curve analyzed with AUC values calculated using the pROC package. DeLong test was conducted for AUC comparisons. Survival analysis was performed using K-M curves with multivariate log-rank test using the survival package. Brier scores were calculated using the risk Regression package. A two-tailed *P*-value <0.05 was considered statistically significant.

## Results

### Baseline characteristics

**Figure 1:**
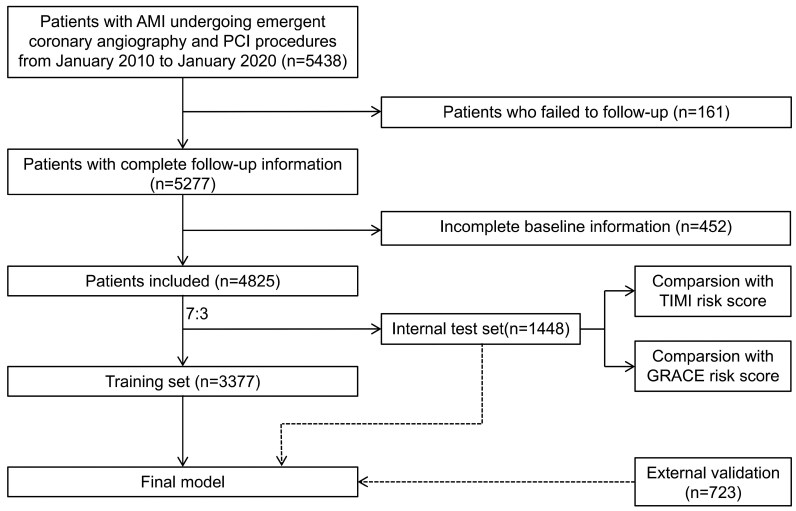
Study flow chart. ACS, acute coronary syndrome; GRACE, Global Registry of Acute Coronary Events (version 2.0); AMI, acute myocardial infarction; PCI, percutaneous coronary intervention; TIMI, Thrombolysis in Myocardial Infarction.

*Figure 1* shows the flowchart of patient selection and study process. After excluding 161 patients lost to follow-up and 452 patients have incomplete baseline information, a total of 4825 patients were included for model development and validation, with a median follow-up period of 28 months (Q1-Q3: 17–58 months). During the follow-up, there were 246 (5.1%) patients died. Baseline characteristics are presented in *Table 2*. The training and test sets included 3377 and 1448 patients, respectively. In the training set, there were 174 (5.2%) cases of mortality, while in the test set 72 (5.0%) cases of all-cause death were recorded. There were no substantial differences regarding the training and test sets (see Supplementary material online, *Table S1*).

**Table 2:** Clinical characteristics of patients

	Overall (*n* = 4825)	All-cause death		
		No (*n* = 4579)	Yes (*n* = 246)	*P*-value
Demographics				
Male, %	3852 (79.7)	3687 (80.5)	165 (67.1)	<0.001
Age, years	59 (51–68)	58 (53–67)	70 (61–76)	<0.001
BMI, kg/m^2^	25.8 (23.6–28.0)	25.8 (23.7–28.0)	25.0 (22.5–27.3)	<0.001
Medical history				
AF	261 (5.4)	228 (5.0)	33 (13.4)	<0.001
Hypertension	2991 (61.9)	2806 (61.3)	185 (75.2)	<0.001
Hyperlipidemia	4427 (91.6)	4209 (91.9)	218 (88.6)	0.074
Diabetes	1577 (32.6)	1478 (32.3)	99 (40.2)	0.012
CKD	342 (7.1)	292 (6.4)	50 (20.3)	<0.001
Stent implantation	697 (14.4)	653 (14.3)	44 (17.9)	0.114
CABG	62 (1.3)	55 (1.2)	7 (2.8)	0.037
Personal history				
Smoking	3261 (67.5)	3124 (68.2)	137 (55.7)	<0.001
Physical examination				
Heart rate, bpm	75 (66–86)	75 (66–85)	80 (72–93)	<0.001
SBP, mmHg	123 (111–136)	123 (111–136)	125 (110–138)	0.744
DBP, mmHg	75 (67–83)	75 (67–84)	73 (65–81)	0.021
EF, %	55 (50–59)	55 (50–59)	50 (41–56)	<0.001
Killip Classification				<0.001
1	4167 (86.3)	4013 (87.6)	154 (62.6)	
2	488 (10.1)	444 (9.7)	44 (17.9)	
3	72 (1.5)	58 (1.3)	14 (5.7)	
4	98 (2.0)	64 (1.4)	34 (13.8)	
Laboratory values				
TG, mmol/L	1.1 (0.9–1.4)	1.1 (0.88–1.36)	1.1 (0.89–1.39)	0.612
HDL-C, mmol/L	1.2 (1.0–1.7)	1.24 (0.95–1.73)	1.22 (0.90–1.62)	0.082
LDL-C, mmol/L	2.7 (2.1–3.3)	2.67 (2.10–3.27)	2.55 (2.05–3.26)	0.226
Lp(a)_ mg/L	180.0 (84.1–356.0)	179.0 (83.7–355.2)	205.3 (98.2–367.8)	0.169
Creatinine, µmol/L	79.7 (68.9−92.1)	79.0 (68.8–91.7)	89.9 (75.9–112.5)	<0.001
eGFR, mL/min/1.73 m^2^	88.4 (72.8–99.2)	89.1 (74.0–99.7)	70.6 (52.1–85.9)	<0.001
hs-CRP, mg/L	7.0 (2.6–11.7)	6.7 (2.6–11.6)	11.1 (4.78–12.8)	<0.001
D-dimer, μg/mL	0.3 (0.2–0.5)	0.3 (0.2–0.5)	0.5 (0.3–0.9)	<0.001
Procedure-related information				
Pre-procedure TIMI flow				0.044
0	3119 (64.6)	2939 (64.2)	180 (73.2)	
1	192 (4.0)	185 (4.0)	7 (2.8)	
2	522 (10.8)	503 (11.0)	19 (7.7)	
3	992 (20.5)	952 (20.8)	40 (16.3)	
Stent implantation	4201 (87.0)	4015 (87.7)	186 (75.6)	<0.001
Thrombus aspiration	1950 (40.4)	1854 (40.5)	96 (39.0)	0.689
IABP	414 (8.6)	361 (7.9)	53 (21.5)	<0.001
Post-procedure TIMI flow				<0.001
0	64 (1.3)	53 (1.2)	11 (4.5)	
1	22 (0.5)	21 (0.5)	1 (0.4)	
2	85 (1.8)	75 (1.6)	10 (4.1)	
3	4654 (96.3)	4430 (96.7)	224 (91.1)	
Criminal lesion information				
Number of diseased vessels				<0.001
1	1205 (24.9)	1172 (25.6)	33 (13.4)	
2	1517 (31.4)	1457 (31.8)	60 (24.4)	
3	2103 (43.5)	1950 (42.6)	153 (62.2)	
Culprit vessel				<0.001
LAD	2148 (44.5)	2037 (44.5)	111 (45.1)	
LCX	688 (14.2)	668 (14.6)	20 (8.1)	
RCA	1876 (38.8)	1778 (38.8)	98 (39.8)	
LM	91 (1.9)	78 (1.7)	13 (5.3)	
Bypass graft	22 (0.5)	18 (0.4)	4 (1.6)	
Reference diameter, mm	3.0 (2.7–3.5)	3.0 (2.7–3.5)	3.0 (2.7–3.5)	0.714
Lesion length, mm	23.0 (16.0–32.0)	23.0 (16.0–32.0)	24.0 (15.8–35.0)	0.627
Diameter stenosis, %	100.0 (99.5–100.5)	100.0 (95.0–100.0)	100.0 (100.0–100.0)	0.001
AHA Classification				<0.001
A	102 (2.1)	94 (2.1)	8 (3.3)	
B1	366 (7.6)	354 (7.7)	12 (4.9)	
B2	716 (14.8)	698 (15.2)	18 (7.3)	
C	3641 (75.3)	3433 (75.0)	208 (84.1)	
In-hospital medications				
Aspirin	4748 (98.3)	4516 (98.6)	232 (94.3)	<0.001
Clopidogrel	3414 (70.7)	3211 (70.1)	203 (82.5)	<0.001
Ticagrelor	1388 (28.7)	1349 (29.5)	39 (15.9)	<0.001
ACEI/ARB/ARNI	3460 (71.6)	3328 (72.7)	132 (53.7)	<0.001
Beta-blockers	4237 (87.7)	4032 (88.1)	205 (83.3)	0.035
Statin	4536 (93.9)	4307 (94.1)	229 (93.1)	0.491
The type of MI				0.241
STEMI	4319 (89.4)	4093 (89.4)	226 (91.9)	
NSTEMI	506 (10.5)	486 (10.6)	20 (8.1)	

Abbreviations: AF, atrial fibrillation; BMI, body mass index; CABG, coronary artery bypass grafting; CKD, chronic kidney disease; DBP, diastolic blood pressure; EF, ejection fraction; eGFR, estimated glomerular filtration rate; HDL, high-density lipoprotein cholesterol; hs-CRP, high-sensitivity C-reactive protein; IABP, intra-aortic balloon pump; LAD, left anterior descending artery; LCX, left circumflex artery; LDL, low-density lipoprotein cholesterol; LM, left main coronary artery; Lp(a), lipoprotein (a); NSTEMI, non-ST-segment elevation myocardial infarction; RCA, right coronary artery; SBP, systolic blood pressure; STEMI, ST-segment elevation myocardial infarction; TG, triglyceride.

External validation cohort consisted of 723 patients with a median follow-up period of 26 months (Q1-Q3: 20–33 months). During the follow-up, there were 29 (4.0%) patients died. Baseline characteristics are presented in Supplementary material online, *Table S2*.

### Model simplification and optimization

#### Selection of machine learning algorithm

Among the 5 ML algorithms, RF displayed the best performance. The C-index of all models on the test set ranged between 0.66 and 0.81. Both the RF and coxPH showed better performance than others (C-index: 0.78 and 0.81, respectively), while the K-M curves of coxPH were not different between low-risk group and median-risk group (*P* = 0.202) (*Table 3*). Thus, we selected the RF model as the concluding risk prediction model. The distribution of risk scores and K-M curves of each model are shown in Supplementary material online, *Figure S2*.

**Table 3:** Comparing the predictive performance of different models

C-index	RF	SSVM	CoxPH	GBSA	XGB
Models trained with all variables					
Train	0.99	0.75	0.82	0.84	0.73
Test	0.78	0.71	0.81	0.76	0.66
Models trained with top 15 variables					
Train	0.97	0.91	0.82	0.82	0.66
Test	0.81	0.70	0.81	0.79	0.56

Abbreviations: CoxPH, Cox Proportional Hazard; GBSA, Gradient Boosting with Stochastic Averaging; RF, Random Forest; SSVM, Survival Support Vector Machine; XGB, XGBoost.

#### Key predictor identification and establish clinical thresholds for continuous variables

SHAP analysis showed that the top 15 variables which contributed most to the model were age, eGFR, D-dimer, creatinine, Killip classification, EF, BMI, heart rate, SBP, hs-CRP, TG, number of diseased vessels, DBP, length of culprit lesion, and stent implantation (see Supplementary material online, *Figure S3*). The optimal clinical thresholds for the continuous variables were as follows: Age = 59.0 years, eGFR = 88.4 mL/min/1.73 m^2^, D-dimer = 0.3 μg/mL, Creatinine = 79.7 µmol/L, EF = 55.0%, BMI = 25.7 kg/m^2^, Heart rate = 75.0 bpm, SBP = 123.0 mmHg, hs-CRP = 6.9 mg/L, TG = 1.1 mmol/L, DBP = 75.0 mmHg, Lesion length = 23.0 mm, Lp(a) = 181.0 mg/L, HDL-C = 1.2 mmol/L, LDL-C = 2.7 mmol/L, Reference diameter = 3.0 mm, and Diameter stenosis = 100.0% (see Supplementary material online, *Figure S4*). Continuous variables were transformed into categorical variables (values below and equate to the cut-off were converted to 0, values above the cut-off were converted to 1) for further analysis.

#### Recursive feature elimination approach

The C-index during RFE is shown in Supplementary material online, *Figure S5*. When the number of variables was reduced to the top 15 with highest SHAP values, the model still maintained excellent predictive performance (C-index = 0.81). However, when the number of variables was reduced to the top 14, the performance of the model became unacceptable (C-index = 0.79). Therefore, we retained the top 15 variables (age, eGFR, D-dimer, creatinine, Killip classification, EF, BMI, heart rate, SBP, hs-CRP, TG, number of diseased vessels, DBP, length of culprit lesion, and stent implantation) with the highest SHAP values for subsequent modelling.

#### Model development and validation (internal and external)

**Figure 2:**
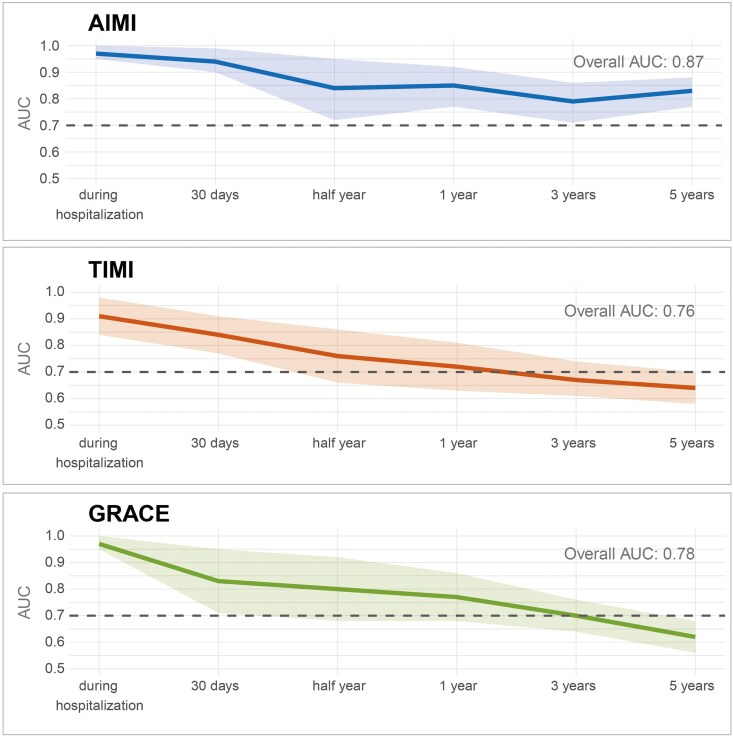
Time-dependent area under the receiver operating characteristic curve (AUC) for three models for all-cause death. AIMI, Artificial Inteligence based prediction model of long-term all-cause death for acute Myocardial Infarction; AUC, area under the curve; GRACE, Global Registry of Acute Coronary Events (version 2.0); TIMI, Thrombolysis in Myocardial Infarction.

We developed the models using the refined set of variables. The RF model was selected as the final model as it performed the best (*Table 3* and Supplementary material online, *Figure S6*). Patients with a risk score <5.09, 5.09 ≤ risk score <14.66, and risk score ≥14.66 were assigned to low-, median- and high-risk groups, respectively. The AIMI model significantly outperformed the GRACE and TIMI risk scores across short-, mid-, and long-term periods in time-dependent AUC analyses (AIMI vs. TIMI, *P* < 0.001; AIMI vs. GRACE, *P* = 0.03, respectively), particularly for predicting long-term all-cause death. The difference between TIMI and GRACE was not statistically significant (*P* = 0.42) (*Figure 2*). Multivariate log-rank test of K-M curves showed the AIMI model discriminates low-, median- and high- risk groups precisely (low-risk vs. median-risk, *P* < 0.001; low-risk vs. high-risk, *P* < 0.001; median-risk vs. high-risk, *P* = 0.001, respectively). In contrast, TIMI and GRACE risk scores could not discriminate the three risk groups very well (TIMI: low-risk vs. median-risk, *P* < 0.001; low-risk vs. high-risk, *P* < 0.001; median-risk vs. high-risk, *P* = 0.46; GRACE: low-risk vs. median-risk, *P* = 0.07; low-risk vs. high-risk, *P* < 0.001; median-risk vs. high-risk, *P* < 0.001, respectively) (see Supplementary material online, *Figure S7*).

**Figure 3:**
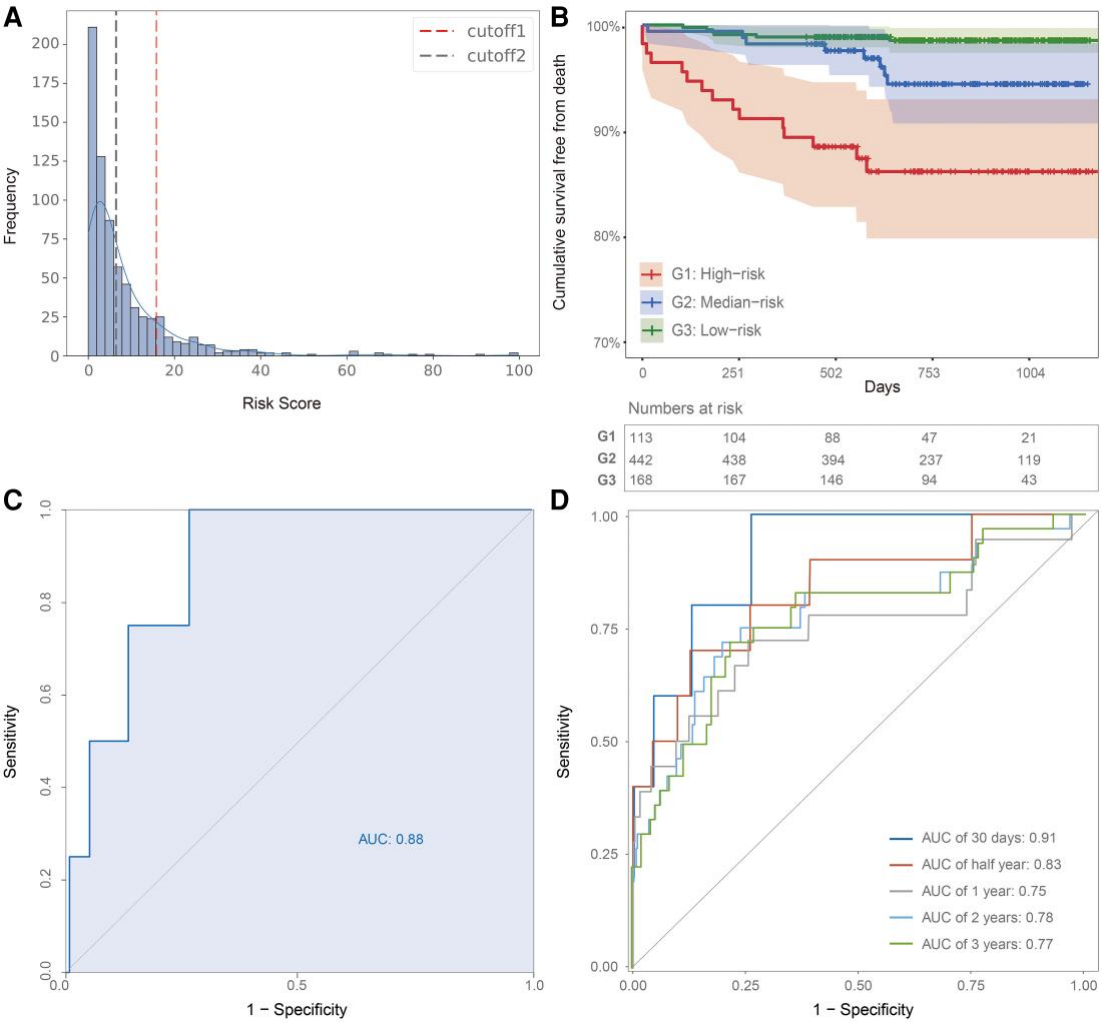
External validation of AIMI model. Risk-score distribution of external cohort (*A*); K-M curves of external validation cohort (*B*); ROC curve during hospitalization of AIMI model in external validation cohort (*C*); ROC curves of 30 days, half year, 1 year, 2 years and 3 years of AIMI model in external validation cohort (*D*).

The model was externally validated (baseline characteristics of the external validation set was shown in Supplementary material online, *Table S2*), with AUC values of 0.88, 0.91, 0.83, 0.75, 0.78 and 0.77 during hospitalization, at 30 days, half-year, 1-year, 2-years and 3-year follow-up, respectively, which suggest a robust generalization capability. Additionally, K-M analysis showed excellent risk stratification performance (high-risk vs. median-risk, *P* = 0.008; high-risk vs. low-risk, *P* < 0.001; median-risk vs. low-risk, *P* = 0.020, respectively) (*Figure 3B*).

The accuracy of AIMI model at a patient level was assessed by Brier scores (*Table 4*). In internal validation cohort, Brier scores showed excellent accuracy at an individual level across different follow-up periods (mean value, 0.022; during hospitalization, 0.003; 30 days, 0.007; half-year, 0.011; 1 year, 0.018; 3 years, 0.039; 5 years, 0.056). This accuracy was also validated in external cohort (mean value, 0.021; during hospitalization, 0.005; 30 days, 0.007; half-year, 0.013; 1 year, 0.024; 2 years, 0.039; 3 years, 0.039).

**Table 4:** Brier scores of AIMI model in internal and external validation cohorts

Brier score	Mean value	During hospitalization	30 days	Half-year	1 year	3 years	5 years
Internal validation	0.022	0.003	0.007	0.011	0.018	0.039	0.056

Brier score	Mean value	During hospitalization	30 days	Half-year	1 year	2 years	3 years
External validation cohort	0.021	0.005	0.007	0.013	0.024	0.039	0.039

### Model interpretation

**Figure 4:**
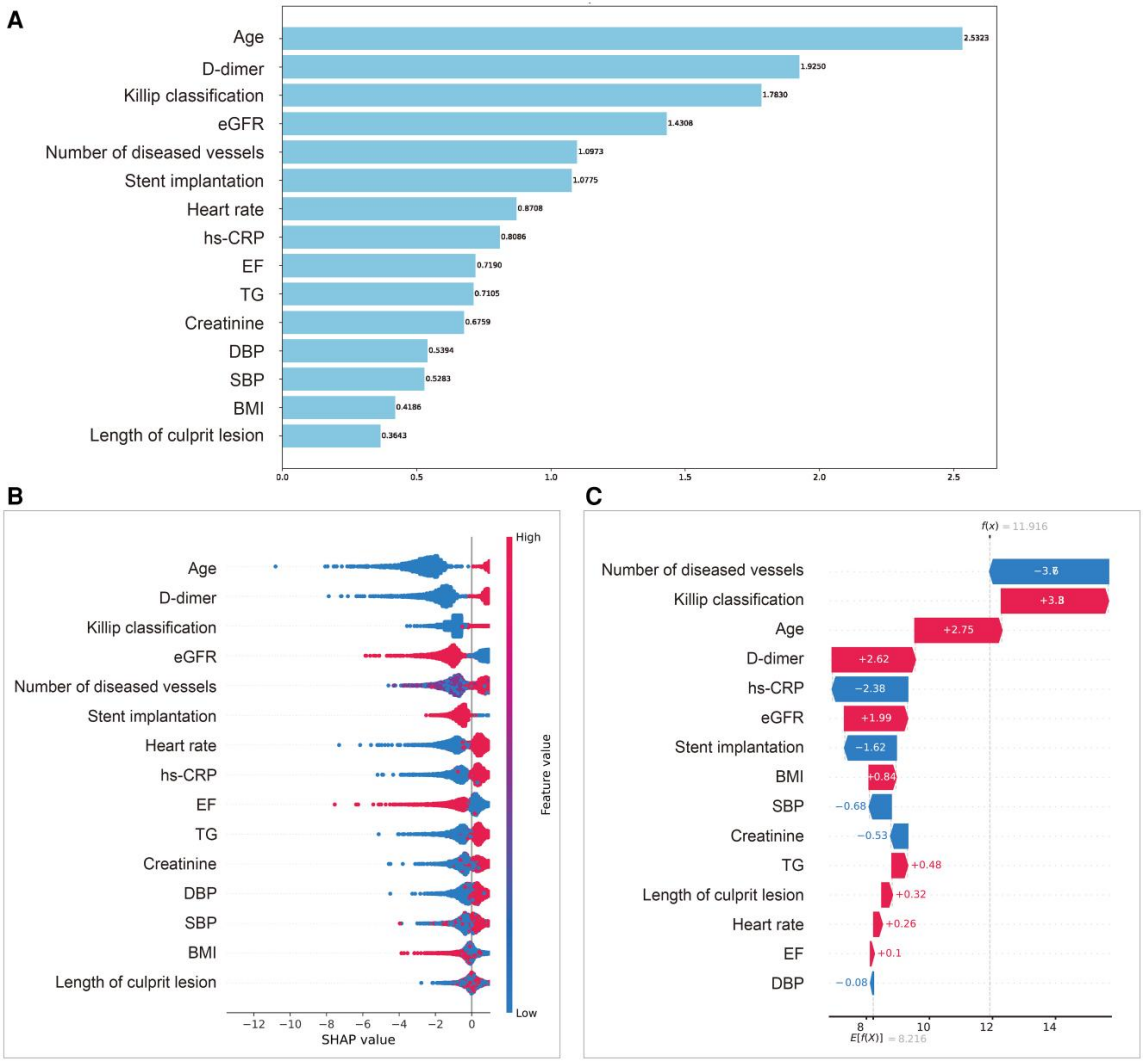
SHAP plot in AIMI model with 15 variables and individualized SHAP plot. SHAP summary plot in population level (*A*, *B*); An example of SHAP plot in individual level (female, 76 years old) (*C*). E[f(X)] represents the mean risk score for all patients and f(x) represents the risk score of each individual patient. BMI, body mass index; DBP, diastolic blood pressure; EF, ejection fraction; eGFR, estimated glomerular filtration rate; hs-CRP, high-sensitivity c-reactive protein; SBP, systolic blood pressure; TG, triglyceride.

At the population level, SHAP plot indicated that higher risk of all-cause death was associated with older age, more diseased vessels, longer lesion length, and higher Killip classification, DBP, SBP, heart rate, D-dimer, hs-CRP, TG and creatinine. Lower risk was related to stent implantation and higher BMI, eGFR and EF (*Figure 4A* and *B*). At the individual level, the AIMI model also provided risk score for each individual patient. Mean risk score for all patients and risk score of each individual were calculated (*Figure 4C*). In contrast to traditional models, the weight of a given feature varies across individual patients.

### Implementation of web calculator

**Figure 5:**
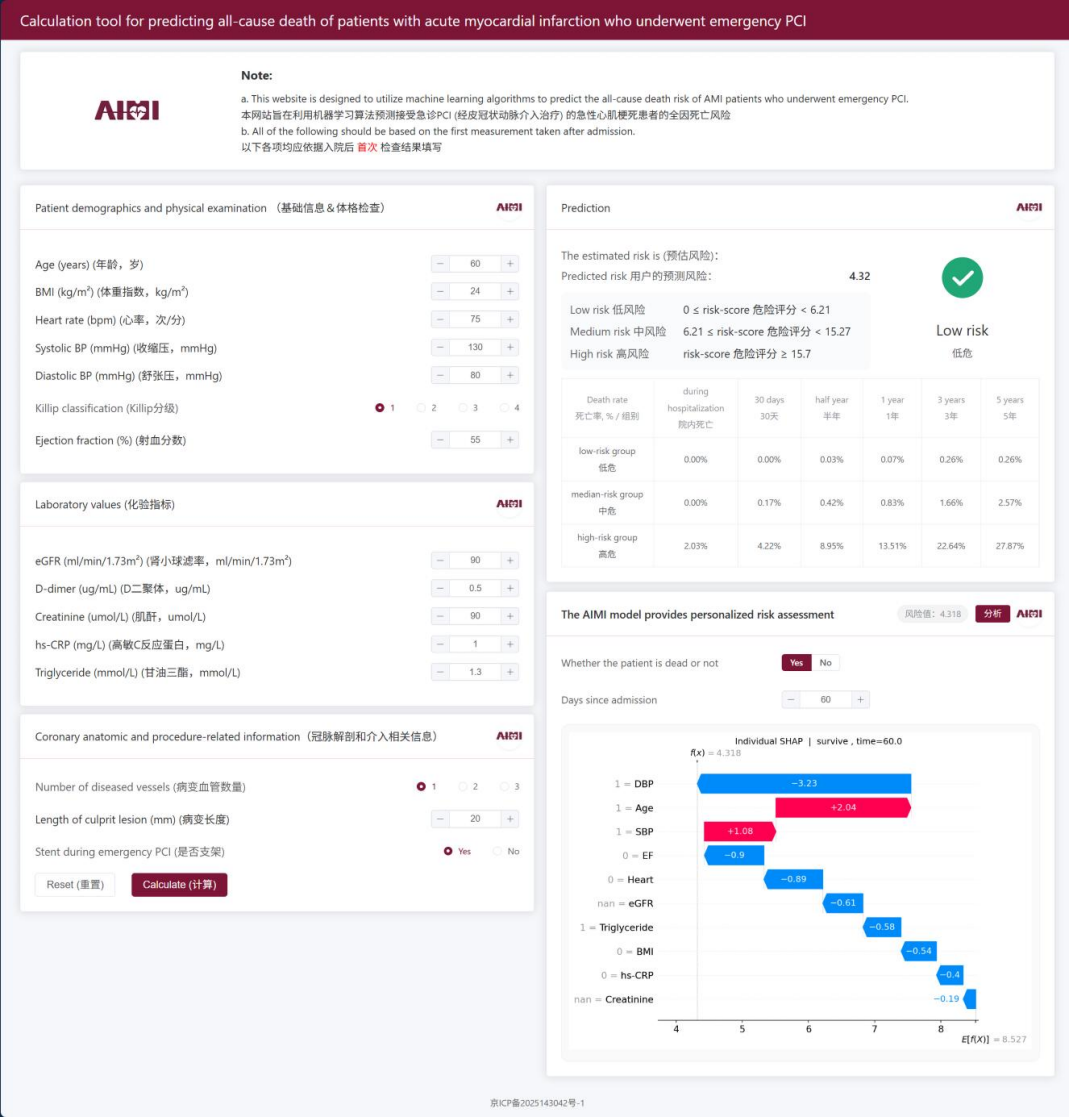
AMI calculator for predicting all-cause death. An example of the web-based tool. A user needs to enter the values first measured during the patient’s admission and click the ‘calculate’ button. The risk score and risk stratification assessed by the model will be shown to the user.

A web calculator was constructed based on these 15 indicators, facilitating individualized prediction of prognostic risk in AMI patients. This calculator provides acute risk assessment and stratification, and assigns different feature weights to individuals (http://aimimodel.com/) (*Figure 5*).

## Discussion

This study demonstrates AI-based model (the AIMI model) by ML is superior to traditional methods for risk stratification in patients after AMI. There are 2 major findings in our study: (ⅰ) the AIMI model showed better performance for predicting all-cause death compared with the GRACE and TIMI risk scores across short- mid- and long-term time points, especially for the long-term outcome; and (ⅱ) a given feature impact varies across individual patients. Although ML-based model has been tested for predicting in-hospital death of patients with AMI,^16^ data are lack for long-term mortality prediction, and the performances of ML-based models are not compared with traditional ones (such as TIMI and GRACE models) regarding long-term outcomes. Additionally, the AIMI model incorporated a more comprehensive set of variables, including more serum biomarkers and coronary anatomic information which were not typically included in previous models, and this contributed to the better performance when compared with traditional risk models. Furthermore, compared with previous AMI prognosis prediction models, AIMI model provides interpretability through SHAP analysis thereby enhancing its transparency and trustworthiness.^17^

### Features differ among the AIMI, TIMI and GRACE models and their clinical availability

Although patients after AMI receive guideline-directed medical therapy (GDMT), including anti-platelet, lipid-lowering and anti-inflammatory therapies and lifestyle interventions, the rate of recurrent ischaemic events remains high, which is called residual risk.^18^ The model identified 15 key features for predicting short- to long-term prognosis of patients with AMI. The common features among AIMI, TIMI and GRACE are age, heart rate, SBP, Killip classification, and serum creatinine.^4,19–21^ The AIMI model also included 10 features not included in TIMI or GRACE models: (ⅰ) there are three procedure-related and coronary anatomic features include stent implantation during PCI, number of diseased vessels and lesion length, which have key impacts on outcome.^22,23^ (ⅱ) the model has four laboratory values include eGFR, hs-CRP, D-dimer and TG. The former 3 features are related to poor prognosis in patients with AMI.^24–26^ Notably, the AIMI model identified TG as a key feature, while LDL-C was not. We speculate that this may be attributed to patients were intensively treated by drugs lowering LDL-C, which is a key target under modern therapy. Although there is no clinical evidence indicates that triglyceride lowering *per se* improves cardiovascular outcomes, an elevated triglyceride level is an independent marker for an increased risk of ischaemic events, and it was considered as marker of residual risk^27^; (ⅲ) the remaining 3 features are EF (instrumental), BMI (demographic) and DBP (physical). Reduced EF is undoubtedly associated with a higher risk of death in patients after AMI.^28^ Patients with higher BMI tended to have a better prognosis, a phenomenon known as the ‘obesity paradox’ [overweight and obese patients with established cardiovascular disease (CVD) exhibit improved prognosis than lean patients with the identical CVD].^29–31^ Low DBP is related to higher mortality after AMI.^32^ Furthermore, Kim *et al.* reported that in AMI patients with multivessel disease who underwent revascularization, an average on-treatment DBP (calculated at admission, discharge and every scheduled visit) ＜ 70 mmHg and ≥ 80 mmHg associated with higher and a tendency of increased long-term all-cause or cardiovascular mortality, respectively. This indicated a J-shaped relationship between DBP and adverse clinical outcomes.^33^ However, in our study, SHAP analysis indicated higher baseline DBP contributes to increased risk of mortality while those with lower baseline DBP did not. We speculate that higher admission DBP may indicate the patient’s blood pressure is not easy to be controlled and thus related to higher long-term risk. Notably, although we initially incorporated medical history, personal history and medications, they were all excluded during feature selection period due to low weights. We speculate that the patients’ compliance was relatively well and thus those factors were relatively well managed.

From a practical perspective, although the AIMI model includes more variables than the TIMI and GRACE scores, these additional features are easily to obtain in modern practice. DBP was a routine index obtained through physical examination. eGFR (calculated through CKD-EPI formula) and BMI are calculated by indicators easily to obtain (creatinine and age for eGFR, height and weight for BMI). Hs-CRP, D-dimer, TG and EF are all conventional indexes commonly examined for patients in China with acute chest pain or diagnosed with ACS. In addition, stent implantation, number of diseased vessels, and lesion length are naturally generated during coronary angiography and PCI procedures, which are necessary for STEMI and NSTEMI patients according to guidelines.^34,35^ Moreover, with current hospital information systems (HIS) and electronic medical records, these variables can also be extracted automatically, which may facilitate implementation of the model in real-world clinical settings. Therefore, the improved predictive performance of AIMI was achieved without substantial additional burden of data collection in the modern era.

### The AIMI model performs better than TIMI and GRACE

Although the GRACE and TIMI scores are well-established models for predicting short-term mortality after AMI,^3,4^ their performance for long-term outcome prediction is relatively weak.^5–7,36^ Our study indicated that the AIMI model has better performance not only for short-term, but also for mid- and long-term risk assessment in AMI patients compared with the TIMI and GRACE risk scores. Moreover, the model was validated in external validation cohorts, verifying its generalization and robustness. We speculate that the following advantages contributed to its superiority. Firstly, our model incorporates more residual risk factors which may have important impacts on prognosis. As mentioned above, features of coronary anatomic and procedure-related information in the AIMI model are well-known factors related to adverse events.^22,23^ Other features, such as triglyceride, eGFR, hs-CRP, D-dimer, DBP and EF may also contribute to the performance of the model.^24–33^ Those features are not included in the TIMI and GRACE risk scores,^37,38^ as the they were originally designed to predict in-hospital and short-term mortality, using features which may be rapidly obtained after patients’ arrival. Secondly, traditional risk scores are established by logistic regression model, which often rely on specific assumptions such as linear relationships between predictors and outcomes,^39^ while AI/ML approach considers interactions between variables and variables’ non-linear and time-dependent influences on outcomes.^36,40^

### The AIMI model provides personalized risk assessment

Although conventional risk stratification tools including TIMI and GRACE risk scores may be useful at the population level, their predictive capacity is more variable at the individual level. We need a more refined approach that allows personalized medical decision-making based on individual patient characteristics. AI/ML has transformative potential in precision cardiology and contributes more personalized risk assessment and prevention strategies.^41^

The strong predictive performance of AIMI model is evidenced by Brier scores, and SHAP plots provide personalized interpretability. During model interpretation, RF algorithm afford risk scores to every patient, and then SHAP analysis assigns different feature weights to individuals based on their risk scores. Individualized SHAP plots help visualize how feature influences vary among individuals (i.e. a given feature impact can be different across individual patients). In addition, the model considers time-dependent influences on outcomes, meaning that feature weights vary at different time points. For example, as AIMI model considered interactions between variables, non-linear and time-dependent influences on outcomes, different weights were assigned to patients at the same age. Those resulted in more accurate risk stratification. Thus, the AIMI model may deliver accurate patient-level predictions with individualized interpretations and has potential for guiding individualized management.

### Clinical value of the improved predictive performance of AIMI

Traditional scores such as GRACE and TIMI are clinically valuable for acute in-hospital risk stratification. These risk scores have proven to be valuable tools for guiding in-hospital management and revascularization strategies. In the overall study population with NSTE-ACS, the benefit of an early invasive strategy vs. a delayed invasive approach remains debated. However, there is a clear signal toward a reduction in MACE with an earlier invasive approach in high-risk patients, particularly those with a GRACE risk score >140.^42,43^ Moreover, when patients were stratified according to the TIMI risk score, intermediate- and high-risk groups derived a significant benefit from an early invasive strategy, with a declined risk of MACE at 6 months.^44^

However, the utility of GRACE and TIMI becomes more limited when moving beyond the acute phase to long-term management.^5–8^ The AIMI model demonstrated better long-term performance. Consequently, AIMI model may help identifying patients at sustained high risk in long-term periods, guiding the selection of long-term treatment strategies beyond the acute phase. Firstly, by identifying patients at persistently high risk and providing individualized risk assessment, AIMI may support closer follow-up and more precise management of residual risk factors. Secondly, the model may help identify patients who remain at sustained high risk beyond the acute phase and may therefore benefit from intensified long-term management strategies, such as stricter lipid-lowering, anti-inflammatory, and anti-thrombotic therapies, as well as better management of comorbidities. Finally, AIMI may also contribute to more efficient healthcare resource allocation by enabling more intensive follow-up for higher-risk patients while reducing unnecessary resource use in lower-risk patients.

Several issues should be addressed in future research. The AIMI model should be evaluated in more diverse populations, including patients from other centres, regions, countries and comorbidity subgroups, to confirm its generalizability and robustness. Moreover, the ultimate value of AIMI lies in whether AIMI-guided management can improve long-term clinical outcomes compared with standard care, which should be tested in future prospective studies.

## Limitations

There are some limitations in our study. Firstly, it is a retrospective study. This may introduce some bias into the results. Secondly, features are not comprehensive enough, lacking information about morphological electrocardiogram features and levels of serum cardiac markers (the data in the early-stage could not be obtained). Thirdly, in external validation cohort, the baseline information [e.g. laboratory values (hs-CRP, d-dimer, TG, creatinine and eGFR) and the coronary anatomic information (lesion length) were not complete enough (especially for patients died during hospitalization) and were filled by RF algorithm, which may reduce the accuracy of the AIMI model]. Finally, although external validation demonstrated the generalization and robustness of AIMI model, the data set is imbalanced, with relatively few events (246 all-cause deaths, 5.1%), which raises concerns about potential model overfitting and instability.

## Conclusions

We established an AI-based model (the AIMI model) by ML for risk stratification in patients after AMI. Internal and external validations showed it performed better than traditional methods such as TIMI and GRACE risk scores in predicting short-, mid-, and long-term all-cause death, especially for long-term all-cause death. AI/ML-based model may have the potential in enhancing risk stratification, guiding clinical decision-making and personalized treatment.

## Supplementary Material

ztag078_Supplementary_Data

## Data Availability

The data underlying this article will be shared on reasonable request to the corresponding author.
