# Supplementary material for: An Artificial Intelligence based model for predicting long-term all-cause mortality after acute Myocardial Infarction (the AIMI model)
Source: Eur Heart J Digit Health. 2026 May 20;7(5):ztag078. doi: 10.1093/ehjdh/ztag078 (PMC13270488; doi:10.1093/ehjdh/ztag078)
Supplement: ztag078_Supplementary_Data [file ztag078_supplementary_data.pdf]

Supplemental Table 1. Baseline characteristics between training set and test set.

	Whole set	Training set	Test set	P-value
**Demographics**				
Male	3852 (79.7)	2716 (80.4)	1136 (78.5)	0.118
Age	59 (51-68)	59 (51-67)	59 (51-68)	0.752
BMI, kg/m²	25.8 (23.6-28.0)	25.7 (23.6-27.9)	25.9 (23.6-28.0)	0.281
**Previous history**				
AF	261 (5.4)	192 (5.7)	69 (4.8)	0.211
Hypertension	2991 (61.9)	2085 (61.7)	906 (62.6)	0.723
Hyperlipidemia	4427 (91.6)	3098 (91.7)	1329 (91.8)	1.000
Diabetes	1577 (32.6)	1108 (32.8)	979 (32.4)	0.789
CKD	342 (7.1)	243 (7.2)	99 (6.8)	0.713
Stent implantation	697 (14.4)	494 (14.6)	203 (14.0)	0.592
CABG	62 (1.3)	45 (1.3)	17 (1.2)	0.780
**Personal history**				
Smoking	3261 (67.5)	2291 (67.8)	970 (67.0)	0.568
**Cardiac physical examination**				
Heart rate, bpm	75 (66-86)	75 (66-86)	75 (66-85)	0.450
SBP, mmHg	123 (111-136)	123 (111-136)	123 (111-135)	0.330
DBP, mmHg	75 (67-83)	75 (67-83)	75 (67-83)	0.884
EF, %	55 (50-59)	55 (50-59)	55 (50-59)	0.319
Killip Classification				0.717
1	4167 (86.3)	2909 (86.1)	1258 (86.9)	
2	488 (10.1)	342 (10.1)	146 (10.1)	
3	72(1.5)	54 (1.6)	18 (1.2)	
4	98(2.0)	72 (2.1)	26 (1.8)	
**Laboratory values**				
TG, mmol/L	1.1 (0.9-1.4)	1.1 (0.9-1.4)	1.1 (0.9-1.4)	0.494

HDL-C, mmol/L	1.2 (1.0-1.7)	1.2 (1.0-1.7)	1.3 (1.0-1.7)	0.930
LDL-C, mmol/L	2.7 (2.1-3.3)	2.7 (2.1-3.3)	2.7 (2.1-3.3)	0.584
Lp(a), mg/L	180.0 (84.1-356.0)	181.0 (83.9-359.3)	175.0 (85.6-350.4)	0.597
Creatinine, μmol/L	79.7 (68.9-92.1)	79.7 (68.8-92.2)	79.6 (69.1-92.0)	0.687
eGFR, mL/min/1.73 m²	88.4 (72.8-99.2)	88.4 (72.8-99.1)	88.5 (72.4-99.9)	0.628
hs-CRP, mg/L	7.0 (2.6-11.7)	6.9 (2.6-11.7)	7.0 (2.8-11.6)	0.903
D-dimer, μg/ml	0.3 (0.2-0.5)	0.3 (0.2-0.5)	0.3 (0.2-0.5)	0.951
**Procedure-related information**				
Pre-procedure TIMI flow				0.110
0	3119 (64.6)	2198 (65.1)	921 (63.6)	
1	192 (4.0)	135 (4.0)	57 (3.9)	
2	522 (10.8)	341 (10.1)	181 (12.5)	
3	992 (20.5)	703 (20.8)	289 (20.0)	
Stent implantation	4201 (87.0)	2953 (61.2)	1248 (86.2)	0.242
Thrombus Aspiration	1950 (40.4)	1379 (40.8)	571 (39.4)	0.370
IABP	414 (8.6)	295 (8.7)	119 (8.2)	0.575
Post-procedure TIMI flow				0.794
0	64 (1.3)	42 (1.2)	22 (1.5)	
1	22 (0.5)	15 (0.4)	7 (0.5)	
2	85 (1.8)	62 (1.8)	23 (1.6)	
3	4654 (96.3)	3258 (96.5)	1396 (96.4)	
**Criminal lesion information**				
Number of diseased vessels				0.898
1	1205 (24.9)	839 (24.8)	366 (25.3)	
2	1517 (31.4)	1059 (31.4)	458 (31.6)	
3	2103 (43.5)	1479 (43.8)	624 (43.1)	
Culprit vessel				0.789

LAD	2148 (44.5)	1496 (44.3)	652 (45.0)	
LCX	688 (14.2)	478 (14.2)	210 (14.5)	
RCA	1876 (38.8)	1320 (39.1)	556 (38.4)	
LM	91 (1.9)	65 (1.9)	26 (1.8)	
Bypass graft	22 (0.5)	18 (0.5)	4 (0.3)	
Reference diameter, mm	3.0 (2.7-3.5)	3.0 (2.7-3.5)	3.0 (2.7-3.5)	0.738
Lesion length, mm	23.0 (16.0-32.0)	23.0 (16.0-32.0)	23.5 (16.0-32.0)	0.760
Diameter stenosis, %	100.0 (99.5-100.5)	100.0 (95.0-100.0)	100.0 (95.0-100.0)	0.776
AHA Classification				0.764
A	102 (2.1)	66 (2.0)	36 (2.5)	
B1	366 (7.6)	260 (7.7)	106 (7.3)	
B2	716 (14.8)	499 (14.8)	217 (15.0)	
C	3641 (75.3)	2552 (75.5)	1089 (75.2)	
**Discharged medications**				
Aspirin	4748 (98.3)	3324 (98.4)	1424 (98.3)	0.803
Clopidogrel	3414 (70.7)	2364 (70.0)	1050 (72.5)	0.084
Ticagrelor	1388 (28.7)	995 (29.5)	393 (27.1)	0.103
ACEI/ARB	3460 (71.6)	2435 (72.1)	1025 (70.8)	0.365
β-blocker	4237 (87.7)	2975 (88.1)	1262 (87.2)	0.362
Statin	4536 (93.9)	3188 (94.4)	1348 (93.1)	0.085
**The type of MI**				0.539
STEMI	4319 (89.4)	3029 (89.7)	1290 (89.1)	
NSTEMI	506 (10.5)	348 (10.3)	158 (10.9)	
All-casue death	246 (5.1)	174 (5.2)	72 (5.0)	0.831

Abbreviations: AF, Atrial Fibrillation; CKD, Chronic Kidney Disease; CABG, Coronary Artery Bypass Grafting; SBP, Systolic Blood Pressure, DBP, Diastolic Blood Pressure; EF, Ejection Fraction; TG, Triglyceride; HDL, High-density Lipoprotein cholesterol; LDL, Low-density Lipoprotein cholesterol; Lp(a), lipoprotein (a); eGFR, Estimated Glomerular Filtration Rate; hs-CRP, high-sensitivity C-reactive protein; IABP, intra-aortic balloon pump; LAD, Left

Anterior Descending artery; LCX, Left Circumflex artery; RCA, Right Coronary artery; LM, Left Main coronary artery; STEMI, ST-Segment Elevation Myocardial Infarction; NSTEMI, Non-ST-Segment Elevation Myocardial Infarction.

Supplemental Table 2. Baseline characteristics of external validation cohort.

	**Overall (n=723)**	**All-cause death**		
		**No (n=694)**	**Yes (n=29)**	**p-value**
**Demographics**				
Male, %	623 (86.17)	601 (86.60)	22 (75.86)	0.172
Age, years	59 (50, 67)	58 (49, 67)	69 (61, 74)	<0.001
BMI, kg/m²	24.79 (22.86, 27.00)	24.80 (22.99, 27.14)	23.20 (22.09, 25.74)	0.033
**Medical history**				
AF	28 (3.87)	21 (3.03)	7 (24.14)	<0.001
Hypertension	396 (54.77)	375 (54.03)	21 (72.41)	0.051
Hyperlipidemia	367 (50.76)	352 (50.72)	15 (51.72)	0.916
Diabetes	279 (38.59)	261 (37.61)	18 (62.07)	0.008
CKD	70 (9.68)	65 (9.37)	5 (17.24)	0.278
Stent implantation	127 (17.57)	120 (17.29)	7 (24.14)	0.342
CABG	8 (1.11)	7 (1.01)	1 (3.45)	0.280
**Personal history**				
Smoking	152 (21.02)	146 (21.04)	6 (20.69)	<.001
**Physical examination**				
Heart rate, bpm	75 (67, 85)	75 (67, 85)	76 (70, 91)	0.210
SBP, mmHg	126 (112, 140)	126 (113, 139)	131 (107, 154)	0.832
DBP, mmHg	77 (70, 85)	77 (70, 85)	76 (67, 80)	0.130
EF, %	56 (49, 61)	57 (50, 61)	52 (36, 60)	0.007
Killip Classification				<.001

1	585 (80.91)	574 (82.71)	11 (37.93)	
2	104 (14.38)	98 (14.12)	6 (20.69)	
3	22 (3.04)	18 (2.59)	4 (13.79)	
4	12 (1.66)	4 (0.58)	8 (27.59)	
**Laboratory values**				
TG, mmol/L	1.59 (1.11, 2.23)	1.59 (1.11, 2.21)	1.52 (1.14, 2.49)	0.739
HDL-C, mmol/L	0.96 (0.83, 1.12)	0.96 (0.83, 1.12)	0.97 (0.79, 1.09)	0.887
LDL-C, mmol/L	2.69 (2.09, 3.41)	2.71 (2.10, 3.42)	2.32 (1.51, 3.06)	0.048
Lp(a)_ mg/L	1.06 (0.97, 1.17)	1.06 (0.97, 1.17)	1.02 (0.97, 1.26)	0.883
Creatinine, μmol/L	87.00 (75.00, 101.00)	87.00 (75.00, 100.00)	104.50 (80.00, 191.00)	0.002
eGFR, mL/min/1.73 m²	84.47 (68.49, 96.94)	84.96 (69.07, 97.44)	65.60 (32.21, 87.26)	<.001
hs-CRP, mg/L	4.34 (1.84, 11.89)	4.31 (1.75, 11.81)	5.64 (2.80, 13.77)	0.480
D-dimer, μg/ml	0.40 (0.26, 0.66)	0.39 (0.26, 0.64)	1.00 (0.41, 1.69)	<.001
**Procedure-related information**				
Pre-procedure TIMI flow				0.663
0	137 (25.28)	130 (24.86)	7 (36.84)	
1	11 (2.03)	11 (2.10)	0 (0.00)	
2	25 (4.61)	25 (4.78)	0 (0.00)	
3	369 (68.08)	357 (68.26)	12 (63.16)	
Stent implantation	624 (86.31)	608 (87.61)	16 (55.17)	<0.001
Post-procedure TIMI flow				0.070
0	9 (2.00)	7 (1.60)	2 (15.38)	
1	2 (0.44)	2 (0.46)	0 (0.00)	
2	9 (2.00)	9 (2.06)	0 (0.00)	
3	430 (95.56)	419 (95.88)	11 (84.62)	
**Criminal lesion**				

**information**				
Number of diseased vessels				0.891
1	157 (21.72)	151 (21.76)	6 (20.69)	
2	218 (30.15)	208 (29.97)	10 (34.48)	
3	346 (47.86)	333 (47.98)	13 (44.83)	
Culprit vessel				0.003
LAD	322 (44.66)	313 (45.23)	9 (31.03)	
LCX	139 (19.28)	134 (19.36)	5 (17.24)	
RCA	238 (33.01)	228 (32.95)	10 (34.48)	
LM	18 (2.50)	14 (2.02)	4 (13.79)	
Bypass graft	2 (0.28)	2 (0.29)	0 (0.00)	
Reference diameter, mm	3.00 (2.50, 3.50)	3.00 (2.52, 3.50)	2.50 (2.46, 3.31)	0.026
Lesion length, mm	27.00 (18.00, 36.00)	27.00 (18.00, 36.00)	28.00 (16.00, 40.00)	0.875
Diameter stenosis, %	99.00 (90.00, 100.00)	99.00 (90.00, 100.00)	99.00 (90.00, 100.00)	0.927
AHA Classification				0.638
A	42 (11.05)	42 (11.54)	0 (0.00)	
B1	68 (17.89)	65 (17.86)	3 (18.75)	
B2	81 (21.32)	77 (21.15)	4 (25.00)	
C	186 (48.95)	177 (48.63)	9 (56.25)	
**In-hospital medications**				
Aspirin	503 (96.73)	490 (98.39)	13 (59.09)	<0.001
Clopidogrel	239 (47.05)	229 (47.31)	10 (41.67)	0.049
Ticagrelor	470 (81.88)	461 (83.36)	9 (42.86)	<0.001
ACEI/ARB/ARNI	503 (83.14)	490 (84.05)	13 (59.09)	0.005
Beta-blockers	653 (95.33)	628 (95.44)	25 (92.59)	0.824
Statin	696 (97.62)	677 (98.69)	19 (70.37)	<0.001

**The type of MI**				0.482
STEMI	346 (47.86)	334 (48.13)	12 (41.38)	
NSTEMI	379 (52.42)	362 (52.16)	17 (58.62)	

Abbreviations: AF, Atrial Fibrillation; CKD, Chronic Kidney Disease; CABG, Coronary Artery Bypass Grafting; SBP, Systolic Blood Pressure, DBP, Diastolic Blood Pressure; EF, Ejection Fraction; TG, Triglyceride; HDL, High-density Lipoprotein cholesterol; LDL, Low-density Lipoprotein cholesterol; Lp(a), lipoprotein (a); eGFR, Estimated Glomerular Filtration Rate; hs-CRP, high-sensitivity C-reactive protein; LAD, Left Anterior Descending artery; LCX, Left Circumflex artery; RCA, Right Coronary artery; LM, Left Main coronary artery; STEMI, ST-Segment Elevation Myocardial Infarction; NSTEMI, Non-ST-Segment Elevation Myocardial Infarction.

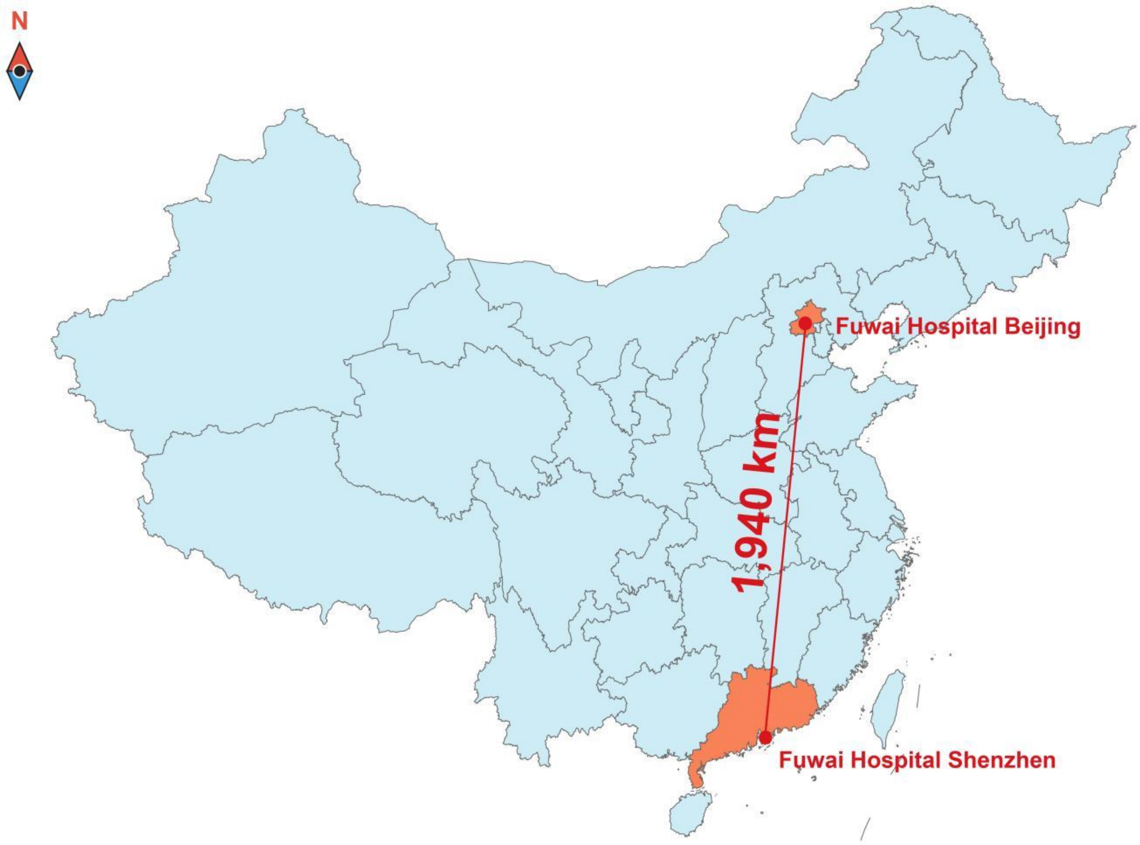

Supplemental Figure 1. The locations of two centers (Fuwai hospital Beijing and Fuwai hospital Shenzhen) in Chinese map.

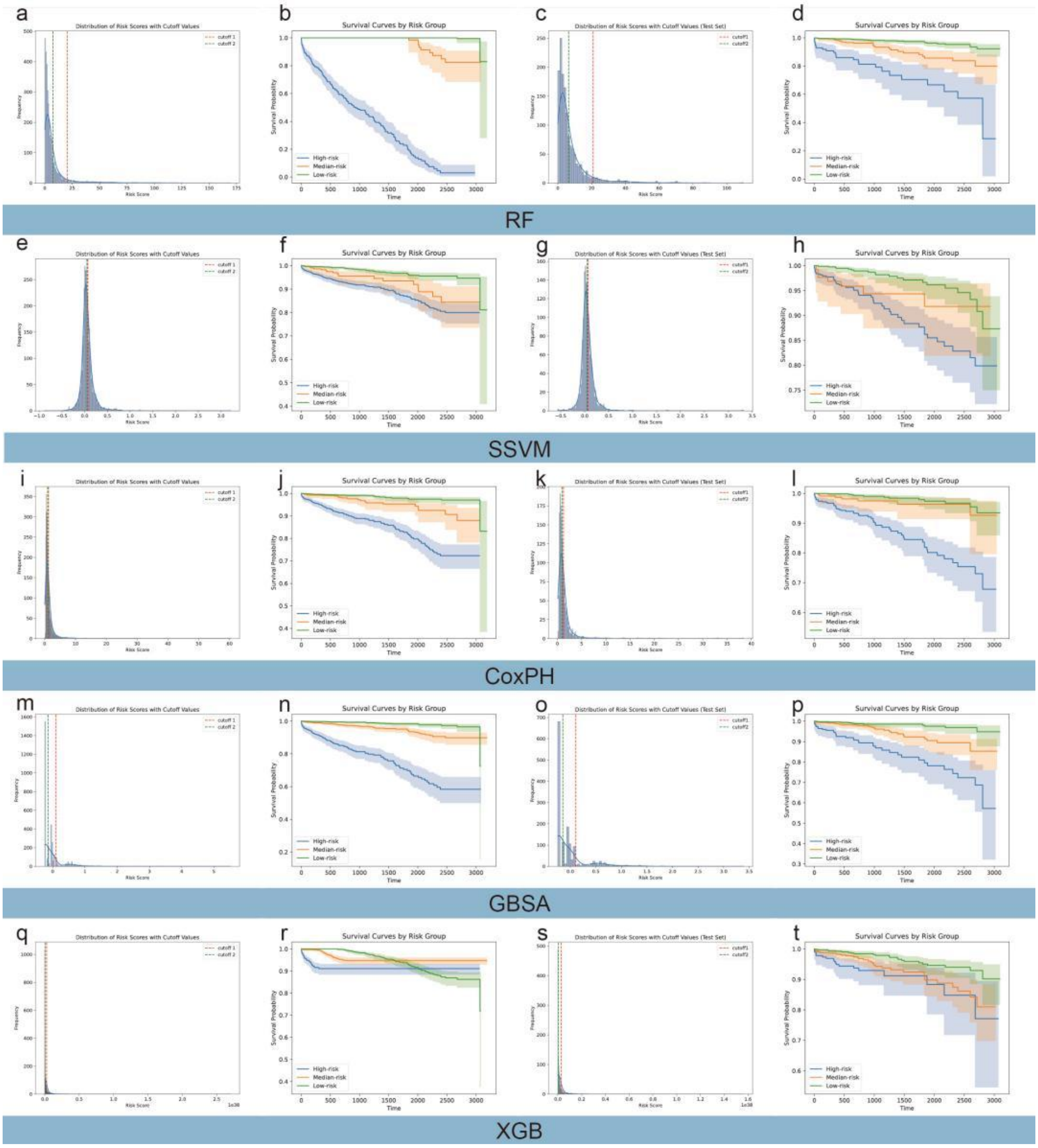

Supplemental Figure 2. The distribution of risk scores and the K-M curves of each model with 42 variables. Distribution of risk scores of training set of RF, SSVM, CoxPH, GBSA and XGB (**a, e, i, m, q)**; K-M curves of training set of RF, SSVM, CoxPH, GBSA and XGB **(b, f, j, n, r)**; Distribution of risk scores of test set of RF, SSVM, CoxPH, GBSA and XGB **(c, g, k, o, s)**; K-M curves of test set of RF, SSVM, CoxPH, GBSA and XGB **(d, h, l, p, t)**.

Abbreviations: RF, Random Forest; SSVM, Survival Support Vector Machine; CoxPH, Cox Proportional Hazard; GBSA, Gradient Boosting with Stochastic Averaging; XGB, XGBoost

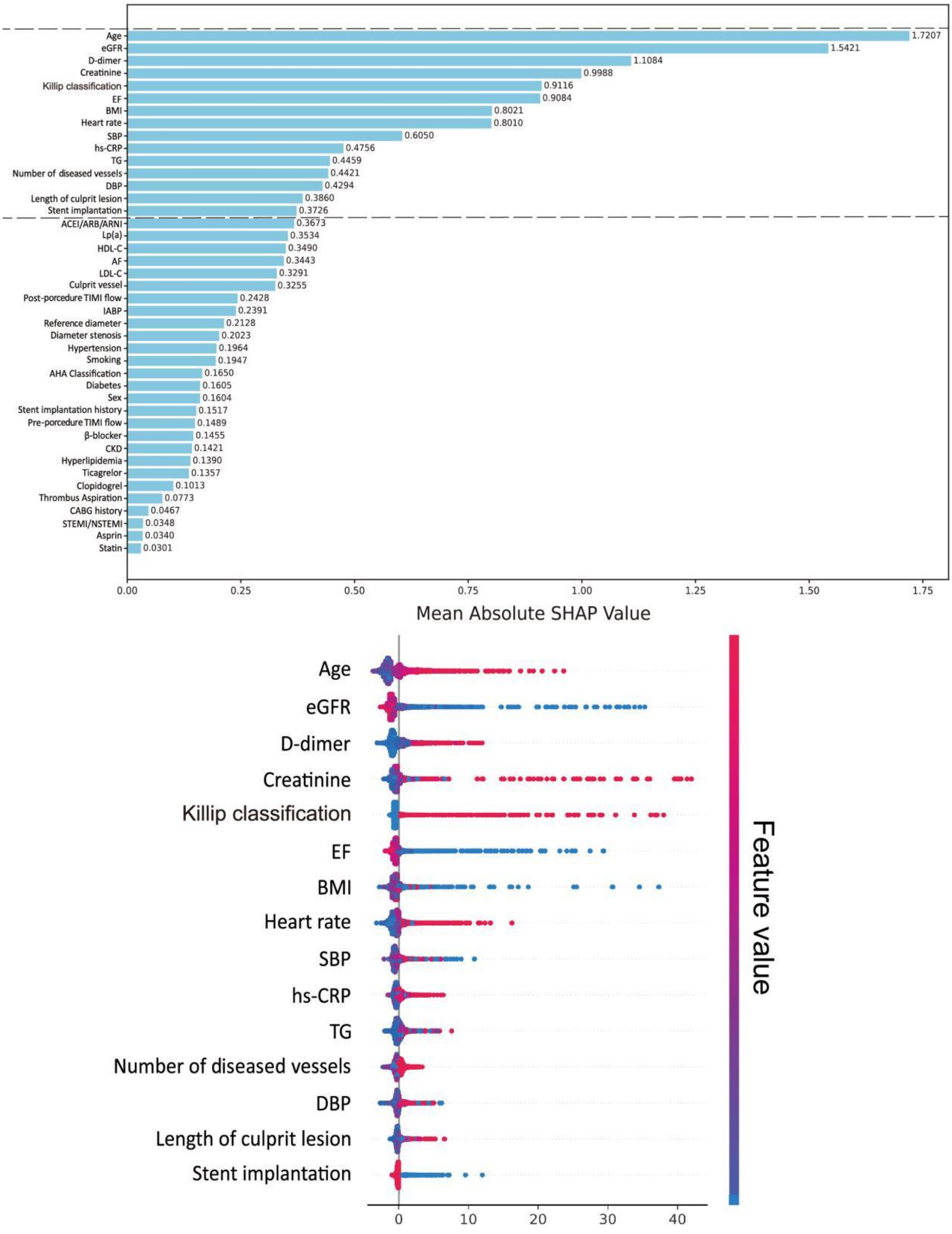

Supplemental Figure 3. SHAP plot in RF model.

Abbreviations: BMI, body mass index; AF, atrial fibrillation; CKD, chronic kidney disease; PCI, percutaneous coronary intervention; CABG, coronary artery bypass grafting; SBP, systolic blood pressure, DBP, diastolic blood pressure; EF, ejection fraction; TG, triglyceride; HDL, high-density lipoprotein cholesterol; LDL, low-density lipoprotein cholesterol; Lp(a), lipoprotein (a); eGFR, estimated glomerular filtration Rate; hs-CRP, high-sensitivity C-reactive protein; IABP, intra-aortic balloon pump; LAD, left anterior descending artery;

LCX, left circumflex artery; RCA, right coronary artery; LM, left main coronary artery; STEMI, ST-segment elevation myocardial infarction; NSTEMI, non-ST-segment elevation myocardial infarction.

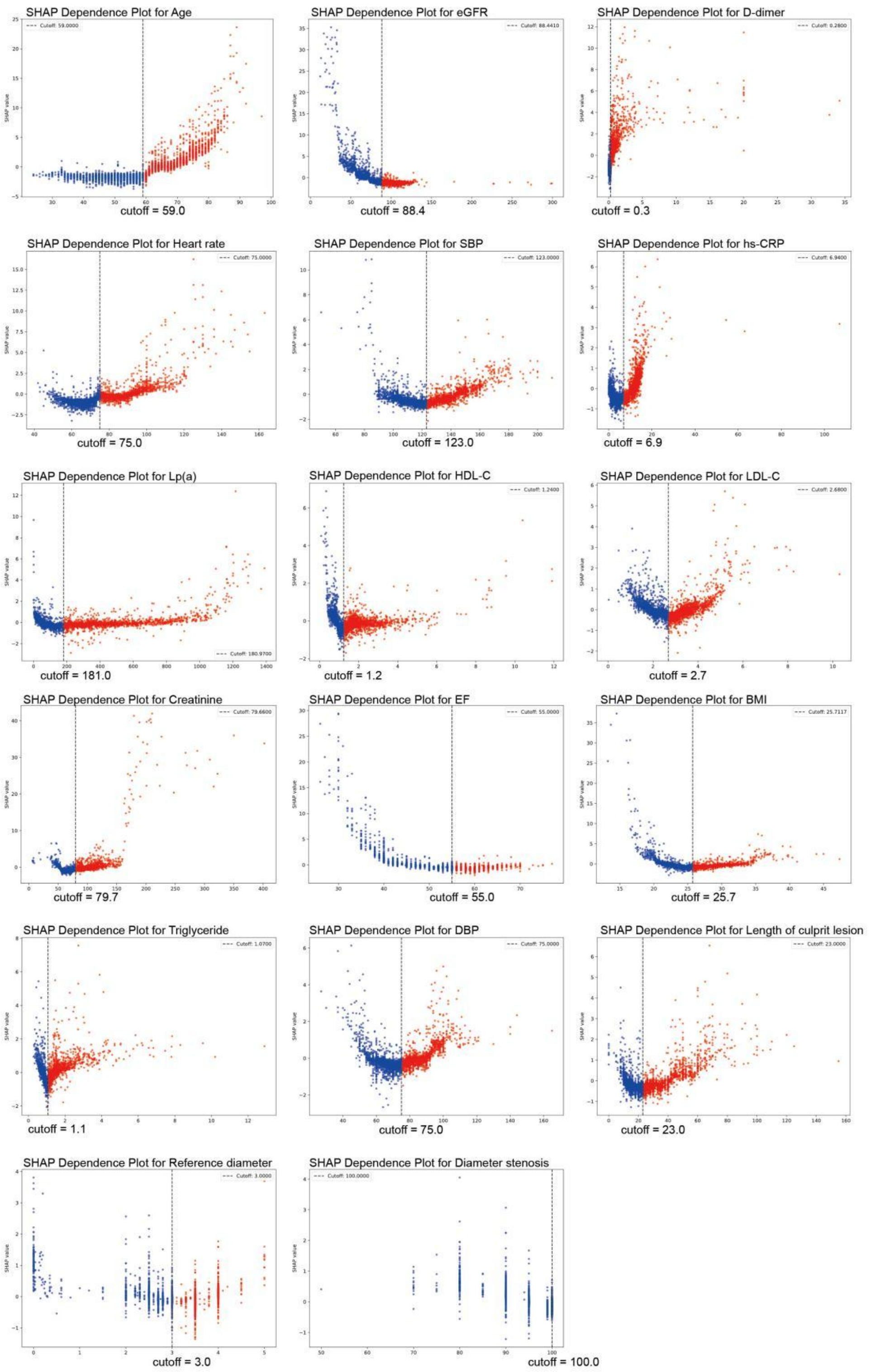

Supplemental Figure 4. The clinical thresholds for the continuous variables.

Abbreviations: eGFR, Estimated Glomerular Filtration Rate; SBP, Systolic Blood Pressure;

hs-CRP, high-sensitivity c-reactive protein; Lp(a), lipoprotein (a); HDL, High-density Lipoprotein cholesterol; LDL, Low-density Lipoprotein cholesterol; EF, Ejection Fraction; DBP, Diastolic Blood Pressure.

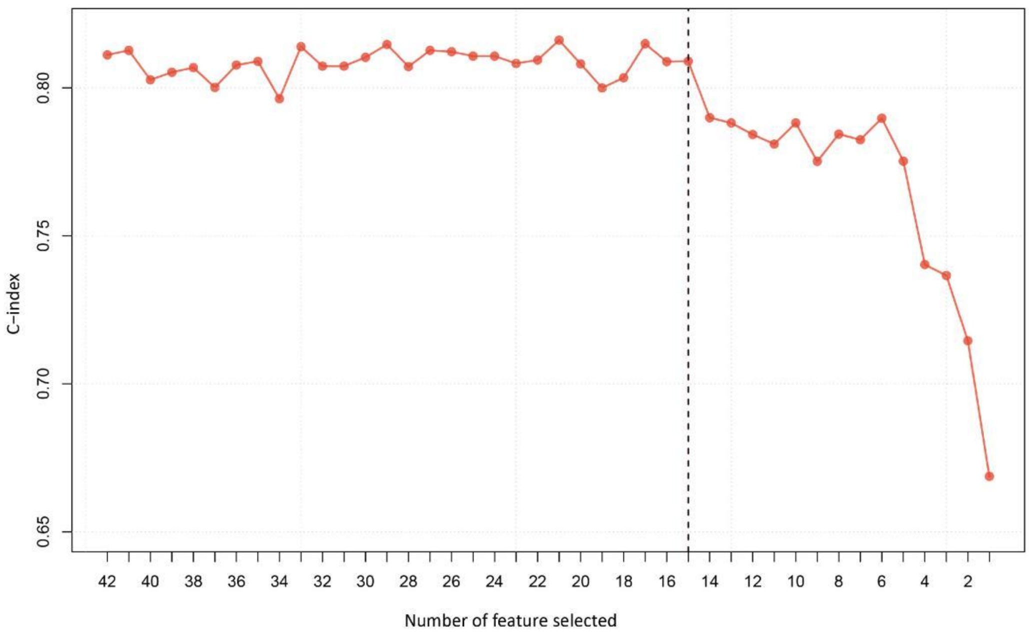

Supplemental Figure 5. C-index during RFE (Recursive Feature Elimination) of RF.

Abbreviations: C-index, concordance index.

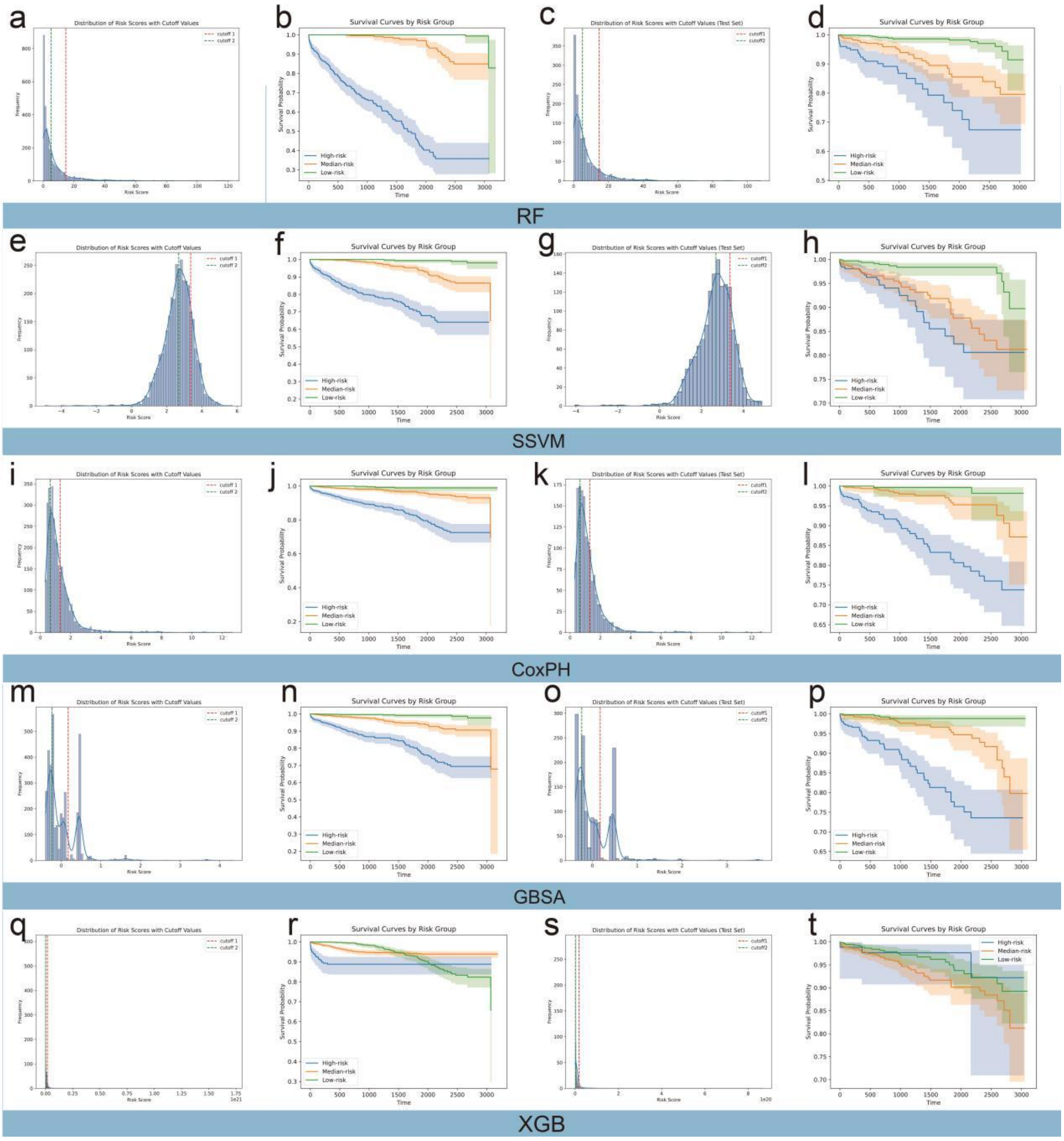

Supplemental Figure 6. The distribution of risk scores and the K-M curves of models with 15 variables. Distribution of risk scores of training set of RF, SSVM, CoxPH, GBSA and XGB (**a, e, i, m, q**); K-M curves of training set of RF, SSVM, CoxPH, GBSA and XGB **(b, f, j, n, r)**; Distribution of risk scores of test set of RF, SSVM, CoxPH, GBSA and XGB **(c, g, k, o, s)**; K-M curves of test set of RF, SSVM, CoxPH, GBSA and XGB **(d, h, l, p, t)**.

Abbreviations: SSVM, Survival Support Vector Machine; CoxPH, Cox Proportional Hazard; GBSA, Gradient Boosting with Stochastic Averaging; XGB, XGBoost

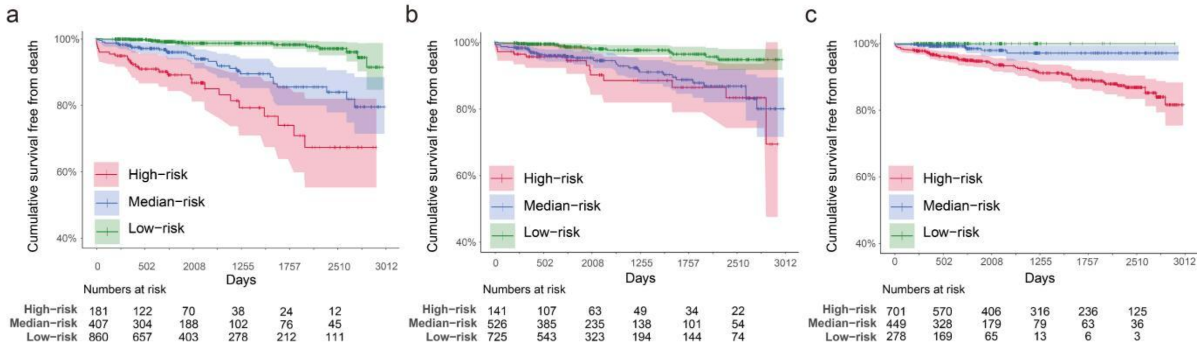

Supplemental Figure 7. The K-M curves of three models. The AIMI model (**a**); The TIMI risk score (**b**); The GRACE risk score (**c**).
